# Efficacy of a new rapid diagnostic test kit to diagnose Sri Lankan cutaneous leishmaniasis caused by *Leishmania donovani*

**DOI:** 10.1371/journal.pone.0187024

**Published:** 2017-11-14

**Authors:** Gayani De Silva, Vijani Somaratne, Sujai Senaratne, Manuja Vipuladasa, Rajitha Wickremasinghe, Renu Wickremasinghe, Shalindra Ranasinghe

**Affiliations:** 1 Department of Parasitology, University of Sri Jayewardenepura, Nugegoda, Sri Lanka; 2 District General Hospital Hambantota, Hambantota, Sri Lanka; 3 Department of Public Health, University of Kelaniya, Sri Lanka; US Food and Drug Administration, UNITED STATES

## Abstract

**Background:**

Cutaneous leishmaniasis (CL) in Sri Lanka is caused by *Leishmania donovani*. This study assessed the diagnostic value of a new rapid diagnostic immunochromatographic strip (CL-Detect^™^ IC-RDT), that captures the peroxidoxin antigen of *Leishmania* amastigotes.

**Methodology/Principal findings:**

We sampled 74 clinically suspected CL lesions, of which 59 (79.7%) were positive by PCR, 43 (58.1%) by Giemsa stained slit skin smear (SSS) and 21 (28.4%) by the new IC-RDT. All samples which were positive either by SSS or IC-RDT or both were positive by PCR. The sensitivities of the IC-RDT and SSS compared to PCR were 36% and 73%, respectively. Fifteen patients from this endemic region were negative by all three tests. Twenty two clinically non-CL skin lesions from a CL non-endemic region were also negative by all three methods. Specificity and PPV of both IC-RDT and SSS compared to PCR were 100%; the NPVs of IC-RDT and SSS were 37% and 58%, respectively. The median parasite grading of the 59 PCR positive samples was 2+ (1–10 parasites/100 HPFs) and IC-RDT positive lesions was 3+ (1–10 parasites /10HPFs). The duration of the lesion was not associated with IC-RDT positivity.

**Conclusions/Significance:**

The median parasite grade of Sri Lankan CL lesions is low. The low sensitivities of SSS and CL Detect^™^ IC-RDT may be due to low parasite counts or low expression of peroxidoxin antigen in amastigotes of the Sri Lankan *L*. *donovani* strain. Our results indicate that negative SSS has to be combined with PCR for confirmation of CL in Sri Lanka. The current commercially available IC-RDT is not suitable to diagnose CL in Sri Lanka; an IC-RDT with improved sensitivity to detect *L*. *donovani* would be a valuable addition in the diagnostic tool kit for Sri Lanka.

## Introduction

Cutaneous leishmaniasis (CL) is a globally important neglected disease of poverty, causing scarring, disfigurement and stigmatization [1], often in the poorest and most marginalized communities [2]. Emergence of new CL foci from different parts of the world including from Sri Lanka has been reported [3–5]. The first endogenous case of CL in Sri Lanka was reported in 1992 from the Southern part of the country [6]. As of 2000, there has been an increase in the number of CL cases that have been reported; initially these cases were seen more commonly among soldiers who were stationed in operational areas in close proximity to forested areas [4,7]. Since then, cases have been reported in other parts of the country and in other communities. One study had reported a significantly higher risk of acquiring CL among people living close to paddy fields than the rest of the community [8]. Another recent study had reported case clustering [9].

Currently, the country has approximately 1200–1500 of new passively detected cases reported per year [7]. The highest numbers of CL cases are reported from Anuradhapura and Hambantota districts, which are geographically separated [10]. Surprisingly, *Leishmania donovani*, a visceralizing species in other parts of the world, is the only identified causative agent of CL in Sri Lanka to date [11–14]. Whole genome sequencing and annotation had shown that the CL causing Sri Lankan strain *L*. *donovani* Mon-37 is a genetic variant of the visceralizing *L*. *donovani* – Mon-37 strain of Sri Lanka and the BPK282A1 visceralizing strain from Nepal [13]. This parasite strain also demonstrated natural attenuation in the BALB/c mouse model of infection [15]. Two studies from Sri Lanka have described *Leishmania* in dogs; one describing the presence of rK39 anti-*Leishmania* antibodies in one out of 141 screened dogs [16] and the other detecting the presence of amastigotes in 1 out of 2 suspected skin lesions in 151 screened dogs and amastigotes in peripheral blood in 1 different dog in the same group of 151 dogs [17]. Whether the Sri Lankan CL causing *L*. *donovani* Mon-37 is a zoonotic or an anthroponotic parasite is yet to be confirmed.

Although CL is becoming a public health problem in Sri Lanka, CL is still commonly diagnosed with traditional slit skin smear (SSS) and biopsy / histology with reported low sensitivities of 35% and 45%, respectively [18]. A recent study reported high sensitivity (92%) and specificity (100%) with a PCR based diagnostic method using pre-described Internal Transcribed Spacer 1 (ITS1) and kDNA primers [14, 19, 20] in detecting Sri Lankan CL. However, PCR is challenging to perform, costly and requires well-equipped laboratories with trained staff, all limiting factors for low-income countries and in field settings, including Sri Lanka.

Early diagnosis and treatment of CL will minimize scar formation, and reduce the potential human-reservoir and spread of the disease in a community. Although immuno-chromatographic Rapid Diagnostic Test kits (IC-RDTs) have been used successfully to detect the prevalence of anti-*Leishmania* antibodies in visceral leishmanisis (VL) and mucocutaneous leishmanisis (MCL), they may be of more limited use in detecting Old World CL where the parasites are confined to the skin and evoke a predominantly cell mediated immune response [21]. The rK-39 IC-RDT had shown high sensitivity (>90%) and specificity (>90%) in detecting anti-*Leishmania* antibodies in VL in the Indian subcontinent [22]. The rK-39 RDT sero-prevalence studies conducted in Sri Lanka in immunocompetent patients with *L*. *donovani* causing CL had not shown any evidence of viceralization of parasite or presence of rK-39 anti-*Leishmania* antibodies in serum [4,8]. A sensitive and specific IC-RDT would be a timely addition to the point of care diagnostic tool kit for CL in less equipped clinics and in active case detection field studies. In addition, an antigen detection IC-RDT may also be used to confirm current infection.

Until recently, no tools were available to detect *Leishmania* antigens in clinical samples. A commercially prepared, USA Food and Drug Administration (FDA) approved qualitative *in vitro* IC-RDT that detects peroxidoxin antigen of amastigotes of *Leishmania* in skin lesions has recently been introduced to the market (CL Detect^™^) (*InBios* International Inc.). This kit uses a monoclonal gold conjugated antibody to capture peroxidioxin from the CL cell lysate and then an affinity purified polyclonal antibody to detect captured peroxidoxin [23,24]. Peroxidoxin, a member of the Thiol-specific antioxidant (TSA) family of proteins, is known to be expressed in both *Leishmania* amastigote and promastigote stage parasites [25, 26], where they play a role in protecting the parasite from destruction by oxidative stress within host macrophages, especially via a recently described molecular chaperon activity [25–29].

The performance characteristics of this new IC-RDT had been tested in Tunisia with *Leishmania major* infections [30, 31] with high sensitivity (100%) and specificity (96%). This IC-RDT had also detected *L*. *donovani* peroxidoxin antigen [24]. Due to the advantages of easy performance, rapid availability of results, high reported sensitivities and specificities [30, 31] and relatively low cost compared to PCR, we conducted a study to determine the sensitivity and specificity of the CL Detect^™^ (*InBios*, USA) IC-RDT in detecting *Leishmania* peroxidoxin antigens in *L*. *donovani* CL lesions in Sri Lankan patients.

## Materials and methods

### Sample collection

Skin samples were taken from suspected CL lesions from 74 patients who attended the Dermatology clinic, District General Hospital, Hambantota from November 2014 to July 2015. Clinical suspicion was made based on the dermatological classification as described before [32] (Table 1). Selected patients were above the age of 18 years, had given written informed consent and did not meet the exclusion criteria. Clinical presentations of the lesions were examined, a brief and relevant history of the illness and photographs were taken. All samples were collected by a trained medical officer after cleaning the lesions and the adjacent normal looking skin sequentially with soap and water, normal saline, and 70% ethanol. The samples for the SSS and IC-RDT were taken after applying a topical local anaesthetic gel {Lidocane 2% (w/v) cream} as recommended by the IC-RDT manufacturer. A punch biopsy sample was taken last from each lesion following an intra-lesional lignocaine injection (2%w/v). All samples were taken from the active edge of the lesions as much adjacent to each other until the required sample numbers were obtained.

**Table 1:** Type of lesions.

Type of lesions	Clinically suspected (f)	True positives* (f)
Papule (≤5mm diameter, palpable solid elevation)	3	3
Nodule (>5 mm diameter, palpable elevation)	26	21
Nodular ulcerative (>5 mm diameter, palpable elevation with central ulceration)	5	4
Dry ulcer (destruction of epidermis of skin with central crusting/scaling)	27	23
Wet ulcer (destruction of epidermis of skin with a wet exudate)	4	3
Plaque (flat topped with diameter greater than its height)	7	4
satellite lesion	2	1
**Total**	**74**	**59**

*True positives were considered when the sample became positive by at least one of the laboratory diagnostic methods, f = frequency

### Exclusion criteria

Children below 18 years of age, suspected CL lesions appearing in areas where punch biopsies could not be taken (i.e; eye lid), persons who had travelled to leishmaniasis endemic country at any point of life, persons who did not give written informed consent and debilitated persons were excluded from the study. The clinically suspected samples that could not be parasitologically proven with at least one diagnostic method were excluded from analysis as the diagnosis could not be confirmed.

### Slit skin smears (SSS)

From every patient, two SSSs were prepared from each lesion to have sufficient tissue material to assess the average parasite density [33]. These smears were stained with Giemsa and examined under oil immersion microscopy for the presence of amastigotes. Parasite density was graded from 0 to 6+ by two trained individuals separately according to WHO guidelines [33]: 0—no parasites per 1000 high power fields (HPF: x 1000 magnification); 1+: 1–10 parasites per 1000 HPFs; 2+: 1–10 parasites per 100 HPFs; 3+: 1–10 parasites per 10 HPFs; 4+: 1–10 parasites per HPF; 5+: 10–100 parasites per HPF; and 6+: > 100 parasites per HPF.

### IC-rapid diagnostic test (IC-RDT)

Samples were collected for the IC-RDT with a sterile disposable dental broach supplied by the manufacturer. The needle was inserted for the same distance (1cm; until the end of the screws) in each lesion to have consistent sample sizes. These samples were then subjected to cell lysis for 10 minutes with 3 drops of lysis buffer and subsequent IC-RDT assay was carried out by placing 20μl of the cell lysate on the test strip. The strip was then dipped in three drops of chase buffer. Appearance or absence of the control and test bands was recorded after leaving for the recommended time (30 minutes). Photographs of the test strips were taken. All procedures described here adhered strictly to the manufacturer’s guidelines and were performed at bedside.

### Negative controls of patients

Twenty-two discarded skin biopsy samples from patients who underwent minor surgical procedures (with no evidence of being a CL lesion) at the Casualty Theater in the Colombo South Teaching Hospital, Sri Lanka located in a non-endemic geographic region for CL, were taken as negative controls. All three tests; SSS, PCR and IC-RDT, were also carried out in the negative control samples.

### DNA extraction and PCR

The 2mm diameter punch biopsy sample that was taken from each lesion was kept in 2 ml of 0.9% normal saline in a standard EDTA blood collection tube (k3EDTA, FL Medical, Italy), transported on ice and stored at -20°C until DNA was extracted. These samples were then subjected to DNA extraction using a commercially available DNA extraction kit (Qiagen DNeasy blood and tissue kit). The extracted DNA was kept at 4°C until PCR was carried out. A previously described and locally validated primer pair, LITSR (Forward) and L 5.8S (Reverse), was selected to amplify a 320 bp fragment of ITS1 region of *Leishmania* genus specific DNA [14, 19, 34]. PCR and gel visualization and capture of photographs were carried out as described elsewhere [14, 19]. DNA extracted from a positive promastigote culture at a parasite count of 1x10^6^/ml was used as a positive control and a no-DNA sample was used as a negative control for PCR in addition to the true negative clinical samples mentioned above.

### Sequencing

Sequencing of the 320 bp amplicon of the ITS1 region from 5 randomly selected CL lesions DNA were carried out at a commercial company (Macrogen, Korea) to confirm these CL causing species in Sri Lanka. All 5 samples with ITS1 320 bp sequence were consistent with *L*. *donovani* with 98–100% score with NCBI-BLAST and were deposited in GenBank (Accession numbers KT273403, KT273404, KT273405, KT273407, KT273408).

### Patient management and follow up

Any clinically suspected patient who was positive by any of the three CL diagnostic tests were considered as proven CL and managed by the consultant dermatologist at District General Hospital, Hamabtota according to dermatological guidelines [35]. The group of clinically suspected but parasitologically negative patients were further investigated for differential diagnoses and managed accordingly by the consultant dermatologist.

### Statistics

Sample size was calculated assuming a sensitivity of 80% and a specificity of 100% for IC-RDT. The standard normal deviation for two sided α with a 95% confidence interval was 1.96. Sensitivity, specificity, positive and negative predictive values (PPV and NPV, respectively) of the IC-RDT test and SSS were compared with PCR. Median parasite grading was also calculated [36]. Parasite densities (grades) were compared with lesion type and duration of the lesions using Chi-square for trend [36] with the programme WINPEPI (PEPI-for-Windows). Detection rate of SSS and IC-RDT were compared with type of lesion, duration of lesion and site of lesion using SPSS version 20.

### Ethics statement

Ethical approval was obtained from the Ethical Review Committee (ERC), Faculty of Medical Sciences, University of Sri Jayewardenepura, Sri Lanka (ERC 787/13). Written informed consent was obtained from every literate participant. A finger print was obtained as consenting to the study from illiterate individuals after reading the form and ensuring that the participants understood the contents contained therein. Because some informed consent was oral (i.e. indicated by thumbprint) the ERC approved the use of thumbprint consent for illiterate participants. All information collected was kept under confidential cover. Further approval to conduct this study including the usage of the IC-RDT as a screening tool for CL was obtained from the Director of the District General Hospital, Hambantota.

## Results

### Clinical features

**Fig 1:**
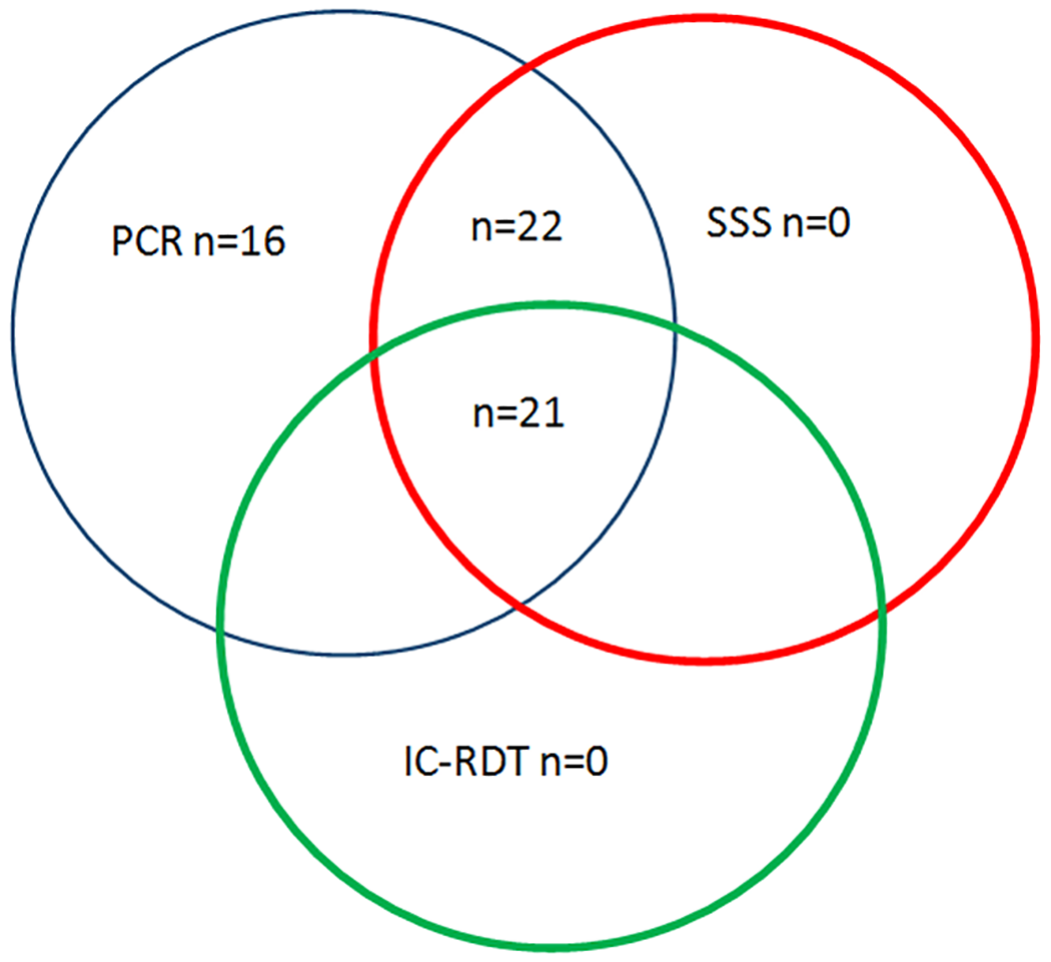
Venn diagram showing the distribution of positive laboratory results. Samples were taken from a total of 74 suspected skin lesions. Out of them only 59 became positive by at least one laboratory test. All three tests were positive only in 21 patients.

The 74 clinically suspected CL patients were between 18–65 years of age. All three tests (SSS, PCR & IC-RDT) were performed in these samples. Out of the tested, only 59 could be confirmed parasitologically positive by at least one laboratory test and could therefore be considered as true positives (PCR positive n = 59, SSS positive n = 43, IC-RDT positive n = 21) (Fig 1). Since the negative 15 patients could not be confirmed as having CL and there are other differential diagnoses for similar lesions (i.e. cutaneous tuberculosis, leprosy and other non-infectious causes i.e sarcoidosis), these patients were excluded from the analysis.

A broad range of clinical presentations were observed from the suspected CL lesions varying from papules, nodules, ulcerative nodules & ulcers to plaques (Table 1). The most frequent clinical presentations were nodules and ulcers (86%). The duration of lesions varied from 1 to 24 months. However, the majority (n = 52/59, 88%) of the lesions had appeared within 1–6 months (Table 2) and all lesions were in exposed areas of the body. The sites of the lesions varied from most commonly in the arm (n = 19), leg (n = 14), forearm (n = 11), face & ear (n = 7) neck (n = 1), abdomen (n = 2), and chest (n = 5). There were 6/59 patients with more than one lesion and the lesion most likely to be a CL lesion was included in the study. These excluded lesions were also tested, reported and appropriately managed by the dermatologist.

**Table 2:** Comparison of different types of lesions with three diagnostic tests.

Lesion type	Duration of lesion	PCR positives	SSS positives	Parasite grade of parasitologically confirmed CL lesions*							RDT positives	Parasite count of RDT positive lesions						
				0	1+	2+	3+	4+	5+	6+		0	1+	2+	3+	4+	5+	6+
Papule	≤4 months	3	2	1		2					2			2				
	5–6 months	-																
	7–12 months	-																
	> 12 months	-																
Nodule	≤4 months	13	11	2	3	5	1	1		1	5			2	1	1		1
	5–6 months	6	4	2	1	2	1				1			1				
	7–12 months	-																
	> 12 months	2	2		1		1				1				1			
^a^Ulcers	≤4 months	17	11	6	4	3	3		1		2				2			
	5–6 months	10	8	2		3	3		2		6			1	3		2	
	7–12 months	3	1	2			1				1				1			
	> 12 months	-																
Plaque	≤4 months	2	1	1				1			1					1		
	5–6 months	1	1			1												
	7–12 months	1	1			1					1			1				
	> 12 months	-																
Other (satellite lesions)	≤4 months	-																
	5–6 months	-																
	7–12 months	-																
	> 12 months	1	1				1				1				1			
**Total**		**59**	**43**	**16**	**9**	**17**	**11**	**2**	**3**	**1**	**21**	**0**	**0**	**7**	**9**	**2**	**2**	**1**

* Parasite grading was done by observing giemsa stained slit skin smears as described in WHO (2010) and parasitological confirmation was done by PCR.

Median of all true positives = 2+, median of all RDT positives = 3+.

^a^ ulcer = (nodular ulcerative +Dry ulcer+ wet ulcer grouped together).

### Validation of IC-RDT

Positive and negative controls provided by the manufacturer with each set of IC-RDT kits were tested before using each pack of kits. Furthermore, to assess the minimal effective concentration to obtain a positive band with the IC-RDT with the Sri Lankan *L*. *donovani* strain, we made tenfold descending serial dilution from 4 x10^3^ to 4x 10^−1^ promastigotes/ml from *in vitro* cultures. A volume of 20 μl from each dilution containing 80 to 0.8 parasites respectively were placed on the IC-RDT and appearance of bands were observed. We observed a detection sensitivity of between 80–8 promastigote equivalents of peroxidoxin.

### Slit skin smear, IC-RDT and PCR

**Fig 2:**
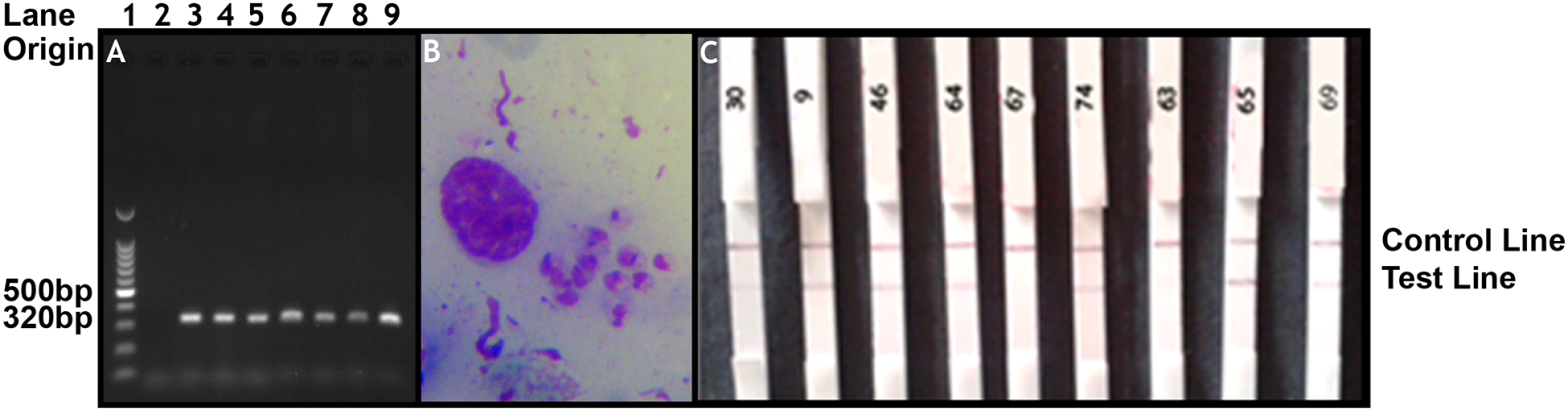
Comparison of laboratory results. (2A) ITS1 PCR result of n = 6/59 samples. Lane 1:100bp DNA ladder, Lane 2: negative control, Lane 3: sample 67, Lane 4: sample 65, Lane 5: sample 63, Lane 6: sample 64, Lane 7: sample 74, Lane 8: sample 73, Lane 9: positive control. (2B) Slit skin smear image. Characteristic amastigotes (sample 64; *grade 4+) x1000 magnification. (2C) Tested IC-RDT strips. Left to right; Strip 1: sample 30 (*grade 2+), Strip 2: sample 9 (*grade 4+), Strip 3 = sample 46 (*grade 3+), Strip 4 = sample 64 (*grade 4+), Strip 5 = sample 67 (*grade 2+), Strip 6 = sample 74 (*grade 2+), Strip 7 = sample 63 (*grade 2+), Strip 8 = sample 65 (*grade 3+), Strip 9 = sample 69 (*grade 3+). *grade = parasite grade [33].

Out of the 59 PCR positive patients, only 43 (73%) subjects were positive for the presence of *Leishmania* amastigotes by SSS. Most of the lesions 37/43 (86%) had moderate to low parasite grades ranging from 1+ to 3+ a median parasite grade of 2+ (Table 2). Furthermore, there were 16 lesions which had a 0 parasite grade and negative IC-RDT results but had a positive PCR (Fig 2). There was only one lesion with the highest parasite grade of 6+ (>100 parasites per HPF). This lesion sample was also positive by both IC-RDT and PCR (Table 2). No statistically significant association could be detected between parasite density and lesion type (ulcer vs no ulcer; papule, nodule, plaque and satellite lesions grouped together as no ulcer) (chi-square for trend = 0.040, (DF = 1), *p* = 0.421) or parasite density and duration of lesion (≤6 months vs ≥ 7 months) (Chi-square for trend = 0.015 (DF = 1), p = 0.451).

IC-RDT was positive only in 21 lesions (35%). All these 21 lesions had a parasite grade of ≥2+ with a median of 3+. However, there were 10 lesions with a parasite grade of 2+, 2 lesions with a parasite grade of 3+ and 1 lesion with a parasite grade of 5+ that were negative by IC-RDT. None of the lesions with a parasite grade of 1+ were positive by IC-RDT. All lesions that were positive by IC-RDT (n = 21) were also positive by SSS and PCR (Table 2). All lesions that were positive by either SSS (n = 43) or IC-RDT (n = 21) or both were always positive by PCR (Fig 1).

Duration of lesion (≤4 month’s vs ≥ 4 months) was not significantly associated with detection rates of either CL-Detect^™^ or SSS. Slit skin smear and CL Detect^™^ detection rates were not associated with type or site of lesion (Table 3).

**Table 3:** Comparison of parasite detection rates of slit skin smear and IC-RDT with different variables.

Type of investigation	Positive	Negative	Sensitivity	Total	Chi-square value	DF	P value
**Detection by SSS vs duration of lesion**							
< 4 months	25	10	71% (25/35)	35	0.092	1	0.76
> 4months	18	6	72% (18/24)	24			
**Total**	**43**	**16**		**59**			
**Detection by SSS vs type of lesion**							
Nodules & ulcers	37	14	73% (37/51)	51	0.021	1	0.89
Others: Papule, plaque, satellite lesions	6	2	75% (6/8)	8			
**Total**	**43**	**16**		**59**			
**Detection by SSS vs site of lesion**							
Head & neck	7	1	88% (7/8)	8	1.74	2	0.42
Trunk	4	3	57% (4/7)	7			
Limbs (upper & lower)	32	12	73% (32/44)	44			
**Total**	**43**	**16**		**55**			
**Detection by IC-RDT vs duration of lesion**							
< 4 months	10	25	10/35 (29%)	35	1.85	1	0.17
> 4months	11	13	11/24 (46%)	24			
**Total**	**21**	**38**		**59**			
**Detection by IC-RDT vs duration of lesion**							
Nodules & ulcers	16	35	31% (16/51)	51	2.92	1	0.09
Others; Papule, plaque, satellite lesions	5	3	63% (5/8)	8			
**Total**	**21**	**38**		**59**			
**Detection by IC-RDT vs site of lesion**							
Head & neck	3	5	38% (3/8)	8	0.18	2	0.92
Trunk	2	5	29% (2/7)	7			
Limbs (upper & lower)	16	28	36% (16/44)	44			
**Total**	**21**			**59**			

### Positive and negative predictive values

All 22 samples that were taken from discarded clinically non-CL skin lesions from patients from CL non-endemic region who underwent minor surgery at the casualty theatre, were negative by all three diagnostic methods (Table 4). Therefore, all three tests showed 100% specificity in detecting CL and SSS and IC-RDT showed PPV of 100% compared with PCR. Further analysis showed that IC-RDT had the lowest sensitivity of 36% with a NPV of 37% compared to PCR. The SSS had a sensitivity of 73% and a NPV of 58% compared to PCR (Table 4).

**Table 4:** Analysis of three diagnostic assays for cutaneous leishmaniasis using PCR as the gold standard.

Diagnostic test	True positives	False positives	True negatives^a^	False negatives	Sensitivity	Specificity	PPV	NPV
**SSS**	43	00	22	16	73%	100%	100%	58%
**IC-RDT**	21	00	22	38	36%	100%	100%	37%

PCR positives were taken as total true positives (n = 59).

^a^ = true negatives from clinically non-CL discarded skin lesions collected from a casualty theatre in a non-endemic area for CL.

NPV = negative predictive value,

PPV = positive predictive value.

## Discussion

Cutaneous leishmaniasis is a newly emerged and established disease in Sri Lanka and its burden on the public health system is increasing. Although there are two reports [16,17] on detecting *Leishmania* parasite in dogs in CL endemic areas in Sri Lanka, whether the transmission is anthroponotic, zoonotic or both is not proven so far. Being a vector borne disease with abundance of the vector [37], having a high possibility of an existing sylvatic cycle with zoonotic transmission [16], and in the absence of government implemented control measures, there is a strong possibility of CL spreading in many parts of the country in future.

In this setting, early detection and treatment of CL patients is a priority to minimize scar formation, stigmatization and to potentially reduce transmission. It is also important to note that CL in Sri Lanka mainly affects the rural communities in low resource settings [4, 8, 9]. The widely used rK-39 RDT is of minimal use in diagnosis of CL in Sri Lanka. Therefore, our study aimed to assess the sensitivity and specificity of a newly introduced field based antigen detection rapid diagnostic tool (CL-Detect^™^) to detect CL in Sri Lanka.

Although CL-Detect^™^ IC-RDT, which was first designed to detect *L*. *major* and has reported high sensitivity and specificity (> 90%) in patients with *L*. *major* infection [30,31], we found low sensitivity (36%) and low NPVs (37%) for the Sri Lankan CL *L*. *donvani* samples. This could be either due to low parasite densities (median grade 2+;1–10 parasites/100 HPF) observed with the Sri Lankan CL lesions compared to higher parasite densities (median 3+; 1–10 parasites/10 HPF) reported with *L*. *major* CL lesions [38] or lower expression of peroxidoxin antigen in Sri Lankan *L*. *donovani* CL strain. The limit of detection of *L*. *donovani* WER 378 promastigotes per CL Detect^™^ IC-RDT strip described by the manufacturer requires 374 parasite equivalents per strip to be positive. Also, the manufacturer’s quality assurance data show that different parasite loads are required with different parasite strains to get positive results [24]. The purified peroxidoxin antigen concentration required for a positive result described by the manufacturer is 100μg/ml [24]. In our study, we could detect as little as 8 parasites per strip in one minimal effective concentration test carried out. Furthermore, the amount of peroxidoxin antigen would vary between the promastigote and amastigote stages; it has been shown before that expression of peroxidoxin 1 gene (Pxn1) is higher in the amastigote stage as compared to the promastigote stage but Pxn2 & Pxn3 genes are less expressed in the amastigote stage as compared to the promastigote stage in VL causing *L*. *chagasi* [39–42]. Peroxidoin concentrations or gene expression levels were not assessed in this study.

In our study, CL Detect^™^ IC-RDTs could not detect a parasite count of 1+ (detection rate 0%). Parasite detection rates of IC-RDT for parasite counts of 2+ and 3+ were 41.2% (n = 7 out of 17) and 81.8% (n = 9 out of 11) respectively. For parasite counts >3+, the sensitivity was 83.3% (n = 5 out of 6) (Table 2) showing that the sensitivity of CL Detect^™^ IC-RDT increases with increase of parasite density in the lesion. The median parasite grade of the CL Detect^™^ IC-RDT positive lesions in our samples was 3+ (Table 2). Surprisingly, there were few lesions (n = 3) with high parasite grades (≥3+) with negative IC-RDT results (Table 2). Low expression of the peroxidoxin antigen by some of the parasites could be a reason for the results observed.

It has been shown in a proteomic analysis study that peroxidoxin concentration produced by the CL strain of Sri Lankan *L*. *donovani* MON-37 promastigotes was 2 times lower than the peroxidoxin concentration produced by VL strain of Sri Lankan *L*. *donvani* MON-37 [41]. The low concentrations of peroxidoxin production could be one of the reasons for possible natural attenuation seen with the Sri Lankan *L*. *donovani* CL strain; the others may be the low A2 gene copy number and SNP mutation in the Rag C gene of the Sri Lankan *L*. *donovani* Mon-37 CL strain detected in a previous study [13]. Since peroxidoxin provides protection to the parasite against the oxidative stress of the host, less peroxidoxin production by the parasite indicates higher susceptibility of the parasite to the host immune response. Therefore, lower expression of peroxidoxin antigen could be a reason for low parasite counts observed in the Sri Lankan CL lesions. No studies have been conducted so far to assess the process of natural healing of CL in Sri Lanka. It will be useful to do further investigations in to peroxidoxin antigen in the Sri Lankan CL causing *L*. *donovani* strain.

The clinical spectrum seen in this study is consistent with previous studies [14,18]. This study also showed that the majority of patients presented before 6 months of the onset of the disease, which may be due to increased awareness in the community. The observed low numbers of papules (n = 3) may be due to ignorance of early lesions by the patient due to its small size and asymptomatic nature of CL lesions. The small number of plaques (n = 4) and satellite lesions (n = 1) observed in this study may be due to less common presentations related to different immune responses of patients which needs further investigation. This study did not categorically assess the sensitivity of diagnostic tests with different types of lesions. However, based on the initial observations made by us, a study comparing sensitivity of available diagnostic tools among different categories of clinical entities will be useful.

Although the detection kit literature specifically states to investigate lesions which are less than 4 months old, our results indicate that the detection rate was higher in lesions more than four months old. Slit skin smear and CL Detect^™^ detection rates were not associated with type or site of lesion probably due to the small sample size.

The SSS sensitivity reported in this study was greater than that reported in the previous study [18]. This could be due to better training skills of the sample collection method and better microscopic observer skills in this current study as sample collectors were pre-trained by an expert and SSS examination was carried out in a specialist leishmaniasis centre.

We observed that obtaining samples from the lesions with the dental broach was difficult probably due to fibrosis of lesions. Entanglement of the broach in the lesion was observed with difficulty in withdrawing the broach. Therefore, replacement of the dental broach with a scalpel blade and obtaining scrapings from the lesions may be considered for sample collection for IC-RDT in future. Sample collection with a scalpel blade for IC-RDT was not tested in our study. Improvement of sample collection technique may improve the sensitivity of the IC-RDT. A limitation of this study was the use of a crude method to assess the parasite density due to unavailability of qRT-PCR facilities in our laboratory. Therefore, it will be useful to assess the actual parasite loads with qRT-PCR in future studies. Assessment of the parasite load would be helpful to assess response to treatment. The limited number of publications to compare the parasite densities with other geographic regions in the world was also a limitation. Albeit no significant relationship could be detected between duration and clinical classification of lesions with parasite densities in this study, low and disproportionate numbers of samples from different types of lesions were limitations in arriving at conclusions in correlations between clinical presentations, duration of lesions and parasite loads (Table 2). However, investigating into parasite load and clinical presentations or duration of lesions was not within the objectives of this study. Therefore, it would be important to compare statistically adequate samples to arrive at conclusions related to parasite densities with other clinical variables in future.

In conclusion, the results of the current study do not support use of the CL Detect^™^ IC-RDT test for the detection of Sri Lankan CL, due to its low sensitivity. However, improvement of this method should be supported due to its user friendly and low cost ($4–5 per test) compared to PCR. Development of a highly sensitive and specific IC-RDT to detect Sri Lankan CL remains a priority. Until then it would be useful to first perform a SSS since it has a sensitivity of 73% at a low cost and then to follow up with PCR in clinically suspected SSS negative CL lesions in Sri Lanka to arrive at a definitive diagnosis.

## Supporting information

S1 Fig Prototypical diagram to report flow of participants through the study. (DOCX)

S1 Table Standards for reporting diagnostic accuracy studies. (DOC)

## References

[1] Kassi M, Kassi M, Afghan AK, Rehman R, Kasi PM. Marring leishmaniasis: the stigmatization and the impact of cutaneous leishmaniasis in Pakistan and Afghanistan. PLoS Negl Trop Dis. 2008;2(10):e259 doi: 10.1371/journal.pntd.0000259 18958168 10.1371/journal.pntd.0000259 PMC2569210

[2] Alvar J, Velez ID, Bern C, Herrero M, Desjeux P, Cano J, et al Leishmaniasis worldwide and global estimates of its incidence. PLoS one. 2012;7(5):e35671 doi: 10.1371/journal.pone.0035671 22693548 10.1371/journal.pone.0035671 PMC3365071

[3] Kolivand M, Fallah M, Salehzadeh A, Davari B, Poormohammadi A, Pazoki Ghohe H, et al An epidemiological study of cutaneous leishmaniasis using active case finding among elementary school students in Pakdasht, southeast of Tehran, Iran 2013–2014. J Res Health Sci. 2015;15(2): 104–8 26175293

[4] Siriwardana HV, Thalagala N, Karunaweera ND. Clinical and epidemiological studies on the cutaneous leishmaniasis caused by *Leishmania (Leishmania) donovani* in Sri Lanka. Ann Trop Med Parasitol. 2010;104(3): 213–23. doi: 10.1179/136485910X12647085215615 20507695 10.1179/136485910X12647085215615

[5] Semage SN, Pathirana KP, Agampodi SB. Cutaneous leishmaniasis in Mullaitivu, Sri Lanka: a missing endemic district in the leishmaniasis surveillance system. Int J Infect Dis. 2014;25:53–5. doi: 10.1016/j.ijid.2014.03.1382 24858902 10.1016/j.ijid.2014.03.1382

[6] Athukorale DN, Seneviratne JK, Ihalamulla RL, Premaratne UN. Locally acquired cutaneous leishmaniasis in Sri Lanka. J Trop Med Hyg.1992;95(6): 432–3 1460704

[7] Epidemiology Unit. Weekly Epidemiology Reports. Ministry of Health Sri Lanka. 2009–2016. http://www.epid.gov.lk/web/index.php?option=com_content&view=article&id=148&Itemid=449&lang=en

[8] Ranasinghe S, Wickremasinghe R, Munasinghe A, Hulangamuwa S, Sivanantharajah S, Seneviratne K, et al Cross-sectional study to assess risk factors for leishmaniasis in an endemic region in Sri Lanka. The American journal of tropical medicine and hygiene. 2013;89(4):742–9. Epub 2013/08/07. doi: 10.4269/ajtmh.12-0640 23918217 10.4269/ajtmh.12-0640 PMC3795106

[9] Kariyawasam KK, Edirisuriya CS, Senerath U, Hensmen D, Siriwardana HV, Karunaweera ND. Characterisation of cutaneous leishmaniasis in Matara district, southern Sri Lanka: evidence for case clustering. Pathogens and global health. 2015;109(7):336–43. Epub 2015/09/09. doi: 10.1179/2047773215Y.0000000032 26345305 10.1179/2047773215Y.0000000032 PMC4768624

[10] Districts of Sri lanka. https://en.wikipedia.org/wiki/Districts_of_Sri_Lanka

[11] Karunaweera ND, Pratlong F, Siriwardane HV, Ihalamulla RL, Dedet JP. Sri Lankan cutaneous leishmaniasis is caused by *Leishmania donovani* zymodeme MON-37. Trans R Soc Trop Med Hyg. 2003;97(4): 380–1 15259461 10.1016/s0035-9203(03)90061-7

[12] Nawaratna SS, Weilgama DJ, Wijekoon CJ, Dissanayake M, Rajapaksha K. Cutaneous leishmaniasis in Sri Lanka. Emerg Infect Dis. 2007 7;13(7):1068–70. 18214182 10.3201/eid1307.060773 PMC2878215

[13] Zhang WW, Ramasamy G, McCall LI, Haydock A, Ranasinghe S, Abeygunasekara P, et al Genetic analysis of *Leishmania donovani* tropism using a naturally attenuated cutaneous strain. PLoS Pathog. 2014;10(7):e1004244 doi: 10.1371/journal.ppat.1004244 24992200 10.1371/journal.ppat.1004244 PMC4081786

[14] Ranasinghe S, Wickremasinghe R, Hulangamuwa S, Sirimanna G, Opathella N, Maingon RD, et al Polymerase chain reaction detection of *Leishmania* DNA in skin biopsy samples in Sri Lanka where the causative agent of cutaneous leishmaniasis is *Leishmania donovani*. Mem Inst Oswaldo Cruz. 2015;110(8): 1017–23. doi: 10.1590/0074-02760150286 26676321 10.1590/0074-02760150286 PMC4708022

[15] McCall LI, Zhang WW, Ranasinghe S, Matlashewski G. Leishmanization revisited: immunization with a naturally attenuated cutaneous *Leishmania donovani* isolate from Sri Lanka protects against visceral leishmaniasis. Vaccine. 2013;31(10): 1420–5. doi: 10.1016/j.vaccine.2012.11.065 23219435 10.1016/j.vaccine.2012.11.065

[16] Rosypal AC, Tripp S, Kinlaw C, Hailemariam S, Tidwell RR, Lindsay DS, et al Surveillance for antibodies to *Leishmania* spp. in dogs from Sri Lanka. J Parasitol. 2010;96(1): 230–1. doi: 10.1645/GE-2288 19803542 10.1645/GE-2288

[17] Nawaratna SS, Weilgama DJ, Rajapaksha K. Cutaneous leishmaniasis in Sri Lanka: a study of possible animal reservoirs. Int J Infect Dis. 2009;13(4): 513–7. doi: 10.1016/j.ijid.2008.08.023 19095480 10.1016/j.ijid.2008.08.023

[18] Ranawaka RR, Abeygunasekara PH, Weerakoon HS. Correlation of clinical, parasitological and histopathological diagnosis of cutaneous leishmaniasis in an endemic region in Sri Lanka. Ceylon Med J. 2012;57(4):149–52. doi: 10.4038/cmj.v57i4.5082 23292056 10.4038/cmj.v57i4.5082

[19] El Tai NO, Osman OF, el Fari M, Presber W, Schonian G. Genetic heterogeneity of ribosomal internal transcribed spacer in clinical samples of *Leishmania donovani* spotted on filter paper as revealed by single-strand conformation polymorphisms and sequencing. Trans R Soc Trop Med Hyg. 2000;94(5):575–9 11132393 10.1016/s0035-9203(00)90093-2

[20] Rodgers MR, Popper SJ, Wirth DF. Amplification of kinetoplast DNA as a tool in the detection and diagnosis of *Leishmania*. Experimental parasitology. 1990;71(3):267–75 2170165 10.1016/0014-4894(90)90031-7

[21] Ronet C, Beverley SM, Fasel N. Muco-cutaneous leishmaniasis in the New World: the ultimate subversion. Virulence. 2011;2(6):547–52. doi: 10.4161/viru.2.6.17839 21971185 10.4161/viru.2.6.17839 PMC3260548

[22] Medley GF, Hollingsworth TD, Olliaro PL, Adams ER. Health-seeking behaviour, diagnostics and transmission dynamics in the control of visceral leishmaniasis in the Indian subcontinent. Nature. 2015;528(7580):S102–8. doi: 10.1038/nature16042 26633763 10.1038/nature16042

[23] Inbios International, Inc. CL DetectTM Rapid test FDA cleared. http://www.inbios.com/wp-content/uploads/2016/06/CL-Brochure-06.26.15-updated-FDA-cleared.pdf

[24] Inbios International, Inc. CL DetectTM Rapid Test for Cutaneous Leishmaniasis, Package insert. http://www.inbios.com/wp-content/uploads/2016/06/900159-00-IVD-CL-Detect-Rapid-Test-Package-Insert.pdf

[25] Webb JR, Campos-Neto A, Ovendale PJ, Martin TI, Stromberg EJ, Badaro R, et al Human and murine immune responses to a novel *Leishmania major* recombinant protein encoded by members of a multicopy gene family. Infect Immun. 1998;66(7): 3279–89 9632596 10.1128/iai.66.7.3279-3289.1998 PMC108343

[26] Bayih AG, Daifalla NS, Gedamu L. DNA-protein immunization using *Leishmania* peroxidoxin-1 induces a strong CD4+ T cell response and partially protects mice from cutaneous leishmaniasis: role of fusion murine granulocyte-macrophage colony-stimulating factor DNA adjuvant. PLoS Negl Trop Dis. 2014;8(12):e3391 doi: 10.1371/journal.pntd.0003391 25500571 10.1371/journal.pntd.0003391 PMC4263403

[27] Harder S, Bente M, Isermann K, Bruchhaus I. Expression of a mitochondrial peroxiredoxin prevents programmed cell death in *Leishmania donovani*. Eukaryotic cell. 2006;5(5):861–70. doi: 10.1128/EC.5.5.861-870.2006 16682463 10.1128/EC.5.5.861-870.2006 PMC1459684

[28] Castro H, Teixeira F, Romao S, Santos M, Cruz T, Flórido M, et al *Leishmania* mitochondrial peroxiredoxin plays a crucial peroxidase-unrelated role during infection: insight into its novel chaperone activity. PLoS Pathog. 2011;7(10): e1002325 doi: 10.1371/journal.ppat.1002325 22046130 10.1371/journal.ppat.1002325 PMC3203189

[29] Teixeira F, Castro H, Cruz T, Tse E, Koldewey P, Southworth DR, et al Mitochondrial peroxiredoxin functions as crucial chaperone reservoir in *Leishmania infantum*. Proc Natl Acad Sci U S A. 2015; 17;112(7): E616–24. doi: 10.1073/pnas.1419682112 25646478 10.1073/pnas.1419682112 PMC4343147

[30] Ben Salah A, N B, Zaatour A, Gharbi A, Chen H, Morkowski S, Needham J, et al CL Detect: selection of an optimal sampling device and evaluation of assay performance againt microscopy and *Leishmania* real time PCR. ASTMH; Atlanta, GA 2012.

[31] Ben Salah A, N B, Zaatour A, Gharbi A, Bettaieb J, Ghawar W, Khedher A, et al Clinical evaluation of CL detect TM rapid test for cutaneous leishmaniasis: performance characteristics when compared to smear microscopy at multiple test sites. ASTMH; New Orleans, LA USA 2014.

[32] RJG Rycroft and SJ Robertson (Editors) 2002. A colour handbook of dermatology. Mansons Publishing Limited, 73 Corringngham Road,London Nw11 7 DL, UK

[33] World Health Organization. Control of the leishmaniases. technical report series. 2010 (949). 21485694

[34] El Tai NO, El Fari M, Mauricio I, Miles MA, Oskam L, El Safi SH, et al *Leishmania donovani*: intraspecific polymorphisms of Sudanese isolates revealed by PCR-based analyses and DNA sequencing. Exp Parasitol. 2001;97(1): 35–44. doi: 10.1006/expr.2001.4592 11207112 10.1006/expr.2001.4592

[35] Sirimanna GMP, Seneviratne JKK, Samaraweera ESN, Ranawaka RR, Hulangamuwa CS, de Silva VNH, et al Guidelines on management of leishmaniasis. Sri Lanka College of Dermatologists 2013:7.

[36] Campbell M J and Swinscow T D V. Statistics at square one. 11^th^ edition ISBN-978-1-4051-9100-5. Wiley-Blckwell, John Wiley & Sons Ltd 10 2009.

[37] Gajapathy K, Peiris LB, Goodacre SL, Silva A, Jude PJ, Surendran SN. Molecular identification of potential leishmaniasis vector species within the Phlebotomus (Euphlebotomus) argentipes species complex in Sri Lanka. Parasites & vectors. 2013;6(1):302 Epub 2014/02/07. doi: 10.1186/1756-3305-6-302 24499561 10.1186/1756-3305-6-302 PMC3853795

[38] Spotin A, Rouhani S, Parvizi P. The associations of *Leishmania* major and *Leishmania tropica* aspects by focusing their morphological and molecular features on clinical appearances in Khuzestan Province, Iran. BioMed research international. 2014;2014:913510 Epub 2014/10/16. doi: 10.1155/2014/913510 25317423 10.1155/2014/913510 PMC4181938

[39] Jing BQ, Deng SS, Zhang RG, Zhang J. Comparative proteomic analysis of the promastigotes and amastigotes of *Leishmania donovani*. Zhongguo Ji Sheng Chong Xue Yu Ji Sheng Chong Bing Za Zhi. 2009;27(2):102–6. 19856494

[40] Harder S, Bente M, Isermann K, Bruchhaus I. Expression of a mitochondrial peroxiredoxin prevents programmed cell death in *Leishmania donovani*. Eukaryot Cell. 2006;5(5):861–70. doi: 10.1128/EC.5.5.861-870.2006 16682463 10.1128/EC.5.5.861-870.2006 PMC1459684

[41] McCall LI, Zhang WW, Dejgaard K, Atayde VD, Mazur A, Ranasinghe S, et al Adaptation of *Leishmania donovani* to cutaneous and visceral environments: in vivo selection and proteomic analysis. Journal of proteome research. 2015;14(2):1033–59. Epub 2014/12/24. doi: 10.1021/pr5010604 25536015 10.1021/pr5010604

[42] Barr SD, Gedamu L. Cloning and characterization of three differentially expressed peroxidoxin genes from *Leishmania chagasi*. Evidence for an enzymatic detoxification of hydroxyl radicals. J Biol Chem. 2001;276(36):34279–87. Epub 2001 Jul 3. doi: 10.1074/jbc.M104406200 11438539 10.1074/jbc.M104406200

