# Supplementary material for: Efficacy of a new rapid diagnostic test kit to diagnose Sri Lankan cutaneous leishmaniasis caused by Leishmania donovani
Source: PLoS One. 2017 Nov 14;12(11):e0187024. doi: 10.1371/journal.pone.0187024 (PMC5685575; doi:10.1371/journal.pone.0187024)
Supplement: S1 Fig — (DOCX) [file pone.0187024.s001.docx]

Potentially eligible participants

n=87

Eligible participants

n=74

No index test

N=0, Reason=All included in the study

Excluded: N=13, Reasons: sites not accessible for punch biopsies, did not give consent

Index test

N=74 (Clinically suspected)

Index test positive

N=21

Index test inconclusive

N=15 (excluded as gold standard PCR was negative)

Index test negative

N= 38

True negatives: n-22

No reference standard =0

Reason+ all had positive control line

No reference standard =0, Reason+ all had positive control line, All 21 were +ve by PCR

No reference standard =0

Reference standard

N=15

Reference standard (PCR)

N=21

Reference standard negative

N=0

True negatives n=22

Final diagnosis:

Target condition present n=59 (PCR +ve),

Condition absent = 0

Final diagnosis: Target condition present (false negatives): 38

Target condition absent (True negatives) = 22

Final diagnosis:

Target condition present: n=0

Target condition absent: n=0

PCR negative 15 were excluded from analysis

**Prototypical diagram to report flow of participants through the study**
